# Brillouin Biosensing of Viscoelasticity across Phase Transitions in Ovine Cornea

**DOI:** 10.3390/bios14080371

**Published:** 2024-07-30

**Authors:** Chingis Kharmyssov, Zhandos Utegulov

**Affiliations:** 1 Department of Science, Astana IT University, Astana 020000, Kazakhstan; 2 Department of Physics, School of Sciences and Humanities, Nazarbayev University, Astana 010000, Kazakhstan

**Keywords:** Brillouin light scattering (BLS), viscoelastic properties, corneal biomechanics, phase transitions, thermal treatments, collagen denaturation, refractive surgery, thermokeratoplasty, photorefractive keratectomy, ovine cornea, temperature-dependent properties, mechanical stability, noninvasive biosensing

## Abstract

Noninvasive in situ monitoring of viscoelastic characteristics of corneal tissue at elevated temperatures is pivotal for mechanical property-informed refractive surgery techniques, including thermokeratoplasty and photorefractive keratectomy, requiring precise thermal modifications of the corneal structure during these surgical procedures. This study harnesses Brillouin light scattering spectroscopy as a biosensing platform to noninvasively probe the viscoelastic properties of ovine corneas across a temperature range of 25–64 °C. By submerging the tissue samples in silicone oil, consistent hydration and immiscibility are maintained, allowing for their accurate sensing of temperature-dependent mechanical behaviors. We identify significant phase transitions in the corneal tissue, particularly beyond 40 °C, likely due to collagen unfolding, marking the beginning of thermal destabilization. A subsequent transition, observed beyond 60 °C, correlates with collagen denaturation. These phase transformations highlight the cornea’s sensitivity to both physiologically reversible and irreversible viscoelastic changes induced by mild to high temperatures. Our findings underscore the potential of the Brillouin biosensing technique for real-time diagnostics of corneal biomechanics during refractive surgeries to attain optimized therapeutic outcomes.

## 1. Introduction

The cornea is crucial for the focusing ability of the human eye, contributing to about two-thirds of its optical power. However, serious eye diseases such as keratoconus, corneal ectasia, hyperopia, recurrent corneal erosion, and certain corneal dystrophies directly impair corneal function. To rectify these diseases, it is often needed to locally deliver heat to diseased regions of the cornea through such heat-induced refractive therapies as thermokeratoplasty [1,2,3] and photorefractive keratectomy [4,5]. Thermal treatments reshape the cornea’s curvature and enhance tissue stability by inducing collagen contraction and new cross-linking bonds, which reorganize the collagen fibers into a more stable structure, thereby correcting refractive errors and improving the cornea’s optical properties. These advanced surgeries, including laser-based refractive interventions, heavily depend on comprehensive sensing of the cornea’s mechanical and optical properties at elevated temperatures relevant for these therapies [6,7,8].

From a materials science perspective, the cornea is an organic composite material, with its stroma predominantly composed of densely arranged collagen. Collagen undergoes a thermally triggered phase change from its triple helical structure to a random coil configuration, a transformation that is intimately linked to tissue contraction [9]. The observed phase change from the random coil state to a gel-like condition is often referred to as hyalinization [10]. This process is believed to involve the breaking of covalent or peptide bonds within the collagen molecules. The hyalinized state is thought to be connected with the stress relaxation of the contracted corneal tissue [11].

Historically, the analysis of collagen denaturation has relied on bulk analytical methods, such as differential scanning calorimetry (DSC) [12,13]. Due to the challenges in performing localized thermal measurements and minimizing the material volume analyzed, microthermal local analysis is achieved by contact-based thermally controlled atomic force microscopy [14]. However, the technique is invasive and lacks sensitivity, failing to produce signals at T < 60 °C. To probe biomechanics noninvasively, a Brillouin light scattering (BLS) spectroscopic sensing via the detection of inelastically scattered light from thermal hypersonic acoustic waves propagating in the probed biological media, is typically employed [15,16,17,18], including noncontact monitoring heating-induced phase transitions in globular proteins [18] and flesh tissues [15].

Using BLS microscopy, the corneal biomechanics was thoroughly studied earlier, but at ambient (room temperature) conditions [19], elucidating critical aspects of biomechanical properties of healthy and keratoconus-affected cornea tissues [20,21], including their depth-dependent mechanical properties and the impact of various corneal cross-linking (CXL) protocols on enhancing corneal stiffness [22,23]. Notably, their investigations revealed significantly lower measured Brillouin shifts in keratoconic corneas compared with healthy ones, indicating a lower elastic modulus and, by extension, decreased biomechanical stability [22]. The efficacy of CXL treatments by BLS has provided valuable insights into the biomechanical reinforcement these treatments offer, especially in the anterior portion of the cornea [23].

Our current study focuses on noninvasive optical biosensing of the ovine cornea across heat-driven phase transitions via monitoring its temperature-dependent GHz viscoelastic properties using BLS spectroscopy, which has never been explored before on corneal tissues. This research direction is critical for a deeper understanding of how temperature variations impact corneal biomechanics. Insights gained from this study are poised to influence the development and refinement of therapeutic interventions such as thermokeratoplasty and laser-assisted procedures, which depend on precise thermal modulation of corneal tissues.

Central to this study is the utilization of Brillouin light scattering (BLS) spectroscopy as a biosensing technique that enables noninvasive, real-time assessment of biomechanical properties at a microscopic level. BLS spectroscopy stands out for its unique ability to detect subtle mechanical changes within biological tissues under various physiological and pathological conditions. Unlike traditional biomechanical testing methods, which often require tissue deformation or destruction, Brillouin spectroscopy provides a contactless alternative that preserves tissue integrity. This feature is particularly advantageous in ophthalmic applications where minimal disruption is crucial. The sensitivity of BLS to changes in mechanical properties at different temperatures makes it an ideal tool for monitoring the dynamic viscoelastic behavior of the cornea during thermal treatments. By integrating BLS into our study, we aim to advance the field of corneal biosensing, offering a sophisticated technique that enhances our understanding of tissue mechanics and aids in the development of targeted, efficacious therapeutic interventions.

## 2. Materials and Methods

### 2.1. Cornea Samples Preparation

Whole eyes from approximately 1-year-old ten ewes were acquired within 2 to 4 h postmortem from a local slaughterhouse. These specimens were stored on ice during transportation and prior to experimentation. To enable optical illumination and detection, each whole eye was positioned within a chamber holder and flattened over a plastic dish. Notably, the flattening process did not induce alterations in the mechanical properties of the cornea. This was confirmed through control experiments involving unflattened eyes suspended in a bath of mineral oil [20]. For the BLS measurements, we opted for silicone oil (CAS Number: 63148-62-9, Sigma Aldrich, St. Louis, MO, USA) instead of mineral oil because of its superior temperature stability and neutrality. All experiments were executed within 2 h of receiving the tissue samples. Brillouin measurements were conducted immediately following the respective treatments, with each sample typically requiring approximately 2 h for data collection.

### 2.2. BLS Sensing

For the measurement of longitudinal (compressional) acoustic waves in studied samples, the BLS spectra were collected using a 180-degree backscattering configuration as described in [24]. Incident light of wavelength $\lambda_{i}$ = 532 provided by a Coherent Verdi G2 solid-state laser operating in a single longitudinal mode was employed. The laser beam was focused onto the cornea samples using a 20× objective lens of a confocal microscope (Mitutoyo, WD = 20 mm, NA = 0.42), maintaining an average noninvasive optical power of 10 mW incident at the samples. The inelastically Brillouin scattered light from the samples was collected and focused using a high-quality anti-reflection-coated camera lens with a focal length of 5 cm, followed by a 40 cm lens directing the light onto a 150 μm-diameter input pinhole of an actively stabilized scanning 3 + 3 pass tandem Fabry–Perot interferometer (JRS Scientific Instruments, DR. J.R. Sandercock, Zwillikon, Switzerland). This interferometer, set to a free spectral range of 20 GHz and a finesse of approximately 100, was used for frequency analysis of scattered light. The light transmitted through the interferometer was further directed onto a 700 μm pinhole and detected by a low-dark count photomultiplier tube (≲1 s^−1^), which converted it into an electrical signal for subsequent computer storage and display. The Brillouin intensity measurements are quantitatively presented to reflect the actual counts detected by our equipment. Specifically, each data point represents an intensity value based on 300 counts collected over an acquisition time of 3 min.

## 3. Results and Discussion

### 3.1. Brillouin Spectra of Cornea and Silicone Oil

**Figure 1:**
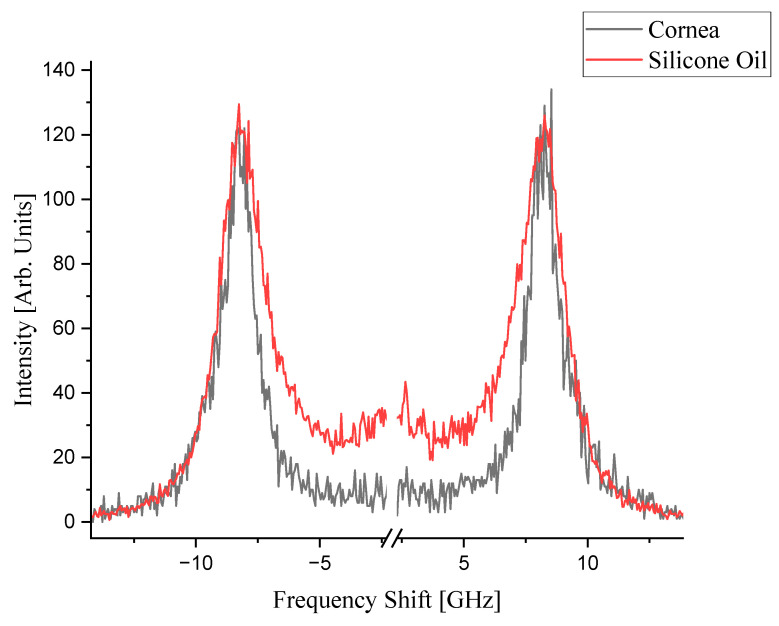
Room temperature Brillouin spectra of the corneal tissue and silicone oil.

The Brillouin spectra collected at room temperature for corneal tissue and silicone oil, as shown in Figure 1, exhibit similar peak shifts that are reflective of the intrinsic mechanical properties of each material. Figure 1 compares the individual spectra of corneal tissue and silicone oil as separate measurements to provide a clear distinction between the two materials without the complexity of overlapping peaks. Direct BLS measurement from dry cornea typically alters its biomechanical properties, as it is significantly influenced by the level of hydration in the cornea [25,26]. Silicone oil was selected for its known and stable viscoelastic properties across various temperatures and immiscibility to water over the studied temperature range [27,28]. This strategy enhances the accuracy of detecting specific biomechanical changes within the cornea, especially under temperature variations, by providing a comparative baseline that underscores the importance of hydration in the accurate assessment of tissue biomechanics.

The corneal tissue presents peaks approximately at ±8.0 GHz, consistent with its known biomechanical properties, while silicone oil demonstrates a distinct line width pattern, which is consistent with [29], evidencing its unique viscous characteristics. During the thermal treatment, the sample was set on a temperature-controlled plate, which was thermally linked to a Peltier device via silver paste (Linkam PE120, Linkam Scientific Instruments, Redhill, UK). This setup was then positioned on a confocal microscope, allowing for measurements to be conducted throughout the heating process. The temperature of the sample was determined based on the temperature voltage calibration of the Peltier element. For subsequent temperature-controlled measurements, the spectra were fitted with multiple Lorentzian functions on the Stokes and anti-Stokes peaks, which yielded peak parameters such as line widths defined as the full width at half maximum (FWHM), frequency peak positions (Brillouin shifts), and integrated peak intensities. The FWHM values for each complex spectral shape were aggregated to quantify the cumulative line widths, capturing the behavior of the samples under varying thermal conditions.

**Figure 2:**
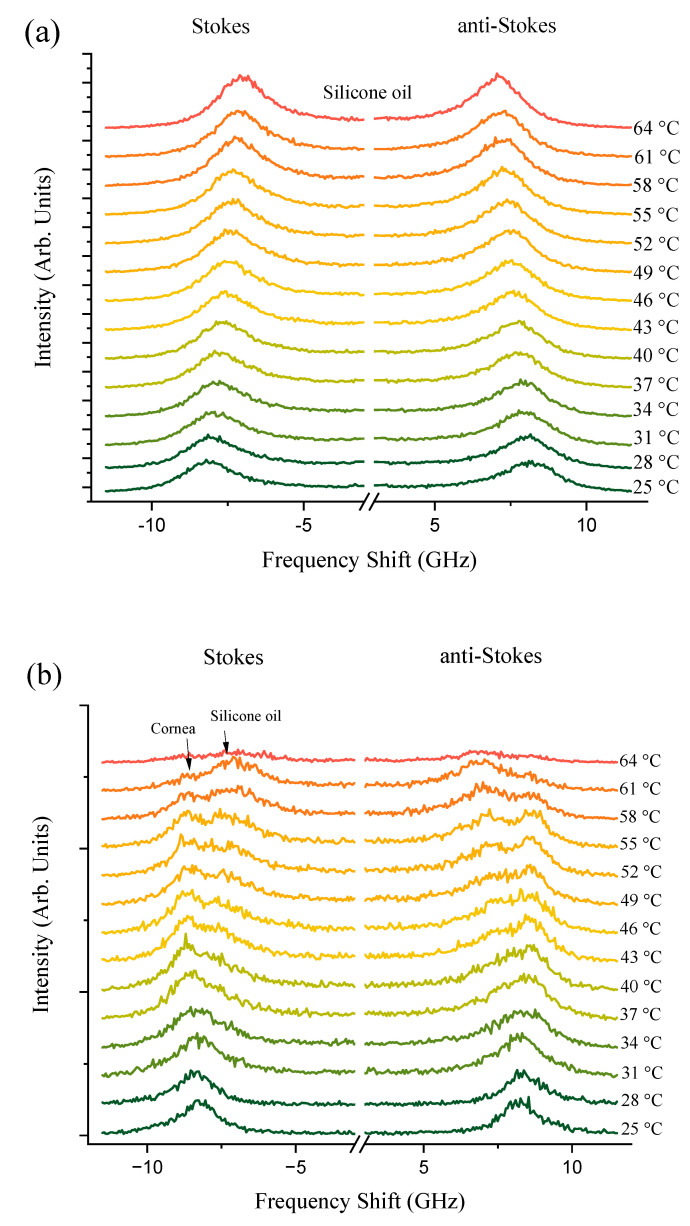
Stokes and anti-Stokes Brillouin spectra of silicon oil (**a**) and cornea (**b**) immersed into silicone oil. Temperatures at which spectra were collected are indicated.

Figure 2a,b present a series of Brillouin spectra of silicone oil and corneal tissue submerged in silicone oil, respectively. This comparison spans a temperature range of T = 25–73 °C. The distinct spectral splitting of the Brillouin peaks observed for silicone oil and corneal tissue at approximately 40 °C suggests the initial thermal destabilization of corneal collagen. This unfolding is in line with the findings of Leikina et al. [30] that Type I collagen undergoes a thermal transition towards a less ordered state at physiological temperatures. Silicone oil, with its viscosity decreasing at higher temperatures, exhibits a more significant shift in Brillouin frequency, corresponding to the decrease in its longitudinal elastic modulus. The corneal tissue, while also affected, shows a lesser frequency shift due to its greater structural rigidity. However, at T > 63 °C, corneal samples become white and opaque, indicative of protein denaturation [31], which leads to significant attenuation of the Brillouin scattering and disappearance of peaks at higher temperatures. These observations are clearly indicative of Brillouin spectroscopy’s sensitivity to biomechanical heat-driven structural phase transformations within the studied biological corneal tissues.

The separation between the corneal and silicone oil peaks is significant as temperature increases, as previously illustrated in Figure 1. This distinction at elevated temperatures is crucial; the sole silicon oil acts as a reference, allowing for a clearer identification of the corneal tissue’s spectral signature even as the thermal effects become more evident. Understanding this separation is pivotal for distinguishing between the Brillouin shifts of the cornea and the silicone oil, which may converge under different experimental conditions. The ability to differentiate between these two materials is of particular importance in high-temperature environments, where the physical properties of the cornea may undergo changes, and the presence of silicone oil can protect and maintain the cornea’s structure.

### 3.2. Phase Transitions: Characteristic Temperatures during Corneal Denaturation

**Figure 3:**
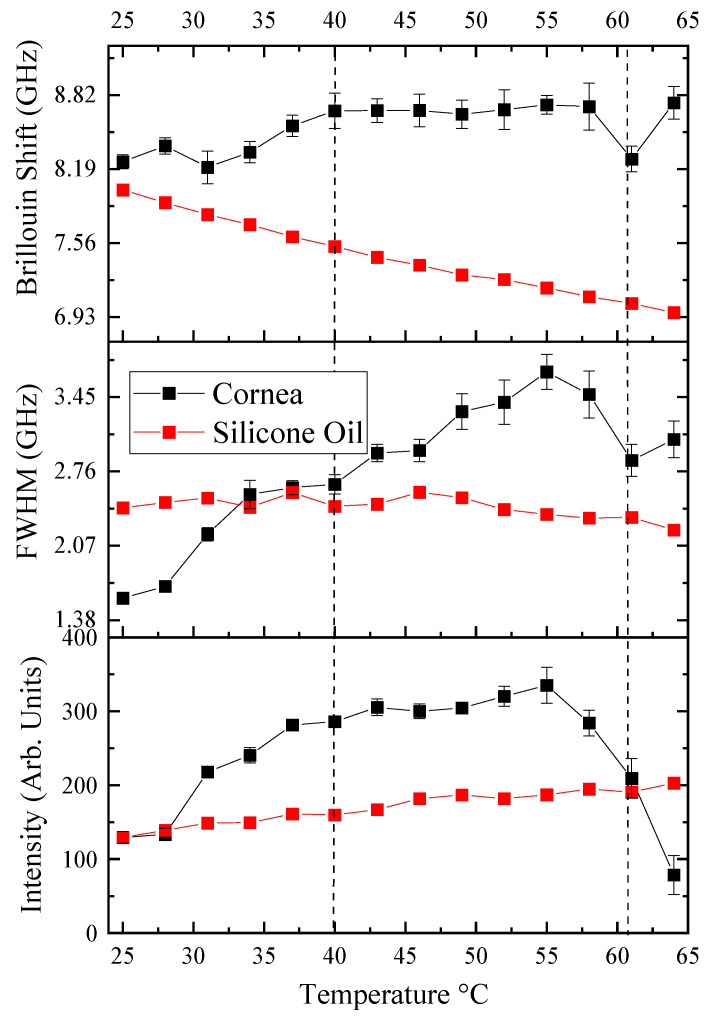
Temperature-dependent BLS spectral parameters of the ovine cornea with temperature-driven phase transition indication with a dashed line.

**Figure A1:**
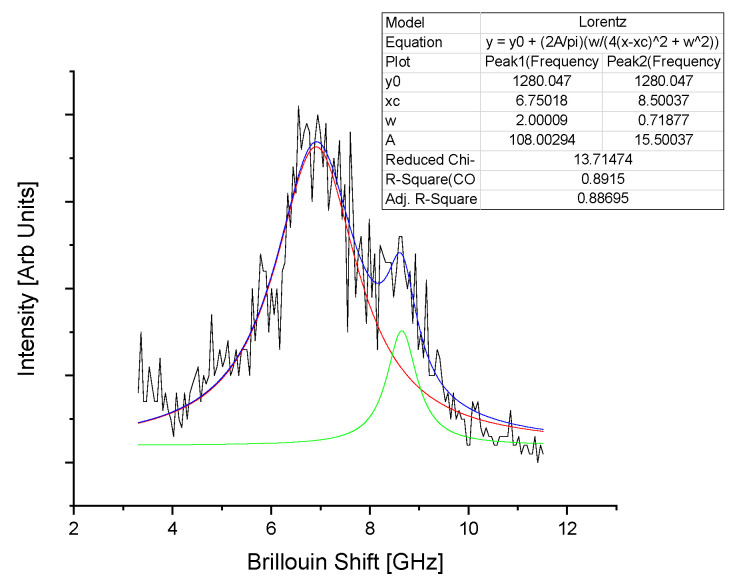
Brillouin spectrum at 64 °C showing multiple Lorentzian fits. The blue line shows the overall fit combining both materials. The red line highlights the contribution from the cornea. The green line shows the contribution from the silicone oil.

Figure 3 presents the raw spectral indicators of phase transitions in ovine corneal tissue as measured by Brillouin Light Scattering (BLS) spectroscopy. This includes the Brillouin shift, Full Width at Half Maximum (FWHM), and intensity, all important markers of the corneal tissue’s biomechanical properties as a function of temperature. It displays the relationship between temperature and the key spectral parameters—Brillouin shift, FWHM, and intensity—each fitted with multiple Lorentzian peaks to reveal the distinct transformations within the tissue’s biomechanical properties as the temperature increases. The figure included in Appendix A (Figure A1) shows a detailed analysis of the Brillouin shift data of a sample at 64 °C. By focusing on these peaks with a refined scale, we demonstrate the presence of two distinct Lorentzian peaks attributed to cornea and silicone oil. In this study, the reported values for silicone oil were derived from Figure 2a, which utilizes a single peak fitting approach. The small standard errors obtained from fitting the central frequency and FWHM across all temperatures indicate precise parameter estimation despite the broader peaks observed. Notably, the increased standard deviation near the cornea’s denaturation temperature (61 °C) reflects the significant physical changes in the tissue, such as opacification and thickening, which contribute to greater measurement variability (Appendix B, Table A1). As the spectral features associated with silicone oil and corneal tissue begin to separate at *T* > 40 °C, a multi-peak Lorentzian fitting approach was implemented using OriginPro software (version: OriginPro 2024b, OriginLab Corporation, Northampton, MA, USA). This nonlinear deconvolution analysis allowed for the precise extraction of peak positions, FWHM, and intensities for the corneal peaks within the composite spectrum, even when overlapping with the silicone oil signal. The Lorentzian fitting parameters were carefully iterated to ensure that the composite fit accurately represented the underlying raw signal. This detailed spectral analysis underpins the observed mechanical behavior changes in the cornea under progressive thermal stress, which is crucial for understanding its viscoelastic and phase transition properties.

Intensity data depicted in Figure 3 highlight significant insights into the biomechanical behavior of ovine corneal tissue under progressive thermal stress. The graph shows a clear peak in the intensity of the Brillouin light scattering signal from the cornea around 50 °C, indicating a critical phase in the thermal response of the tissue. This peak possibly represents the onset of collagen denaturation, where the corneal tissue begins to lose its structural integrity and stiffness. As the temperature continues to rise beyond this peak, the cornea becomes more opaque, as seen by a notable decline in the BLS intensity, i.e., in elasto-optic interaction, reflecting a further degradation of the corneal structure into a more disordered, gelatin-like state. This intensity reduction is consistent with a decrease in the cornea’s ability to scatter light efficiently due to the disruption of its organized collagen matrix [32]. In contrast, the intensity of the silicone oil remains relatively unchanged across the temperature range, underscoring its stability and serving as a control to highlight the thermal sensitivity of the biological tissue.

The Brillouin shift, indicative of the elastic modulus of the cornea, exhibits a pronounced peak at approximately 60–63 °C, signifying a critical change in corneal stiffness. This observation aligns with the anticipated thermal-induced denaturation of collagen, characterized by a transition from the native triple helical structure to a disordered gelatin-like arrangement, resulting in a weakened stromal network [33].

The FWHM values, which reflect the viscoelastic damping properties and acoustic phonon life cycle within the tissue, progressively increase and culminate in a marked surge at this temperature range. This pattern is a clear sign of a phase change where the cornea becomes less stiff and more flexible, which matches up with how the cornea acts when the collagen starts to break down and lose its normal structure. FWHM reveals a progressive rise in the cornea to a phase transition temperature of ~60–63 °C, a pattern that echoes findings in collagen solutions [34] where a temperature elevation led to increased intrinsic viscosity. The temperature range of 60–63 °C corresponds to the denaturation of collagen, where its triple-helical structure unravels into a random coil configuration [35]. This denaturation results in a substantial reduction in the stiffness and viscous behavior of the cornea. The augmentation in the cornea’s viscosity with temperature is attributed to the tendency of collagen molecules within the cornea to aggregate at elevated temperatures, facilitated by increased hydrophobic interactions among the molecules. This aggregation behavior of collagen molecules at elevated temperatures is corroborated by Na et al. [36], who used electrophoresis and thermal denaturation techniques to study molecular properties, and by Pederson et al. [37], who examined thermal responses using X-ray diffraction and calorimetry in their biomimetic studies. Beyond the phase transition temperature (>60 °C), there is an apparent reduction in the cornea’s viscosity. This observation parallels findings in gelatin solutions, where intrinsic viscosity diminishes as the temperature increases [38,39]. The decrease in corneal viscosity at elevated temperatures could be hypothesized to relate to the structural transformation of collagen into gelatin within the corneal tissue. Collagen, the primary structural protein in the cornea, undergoes denaturation at high temperatures, transitioning into gelatin—a process that could lead to the observed reduction in viscosity. This transformation suggests a breakdown or alteration in the collagen’s triple-helical structure, potentially leading to a less organized and, therefore, less viscous state, resembling the behavior of gelatin solutions under similar thermal conditions.

The viscoelastic properties of biological tissues are known to be intricately linked to their microstructural characteristics. In the ovine cornea, the collagen network architecture, which includes factors such as network connectivity, pore size, and fiber diameter, is particularly sensitive to the polymerization temperature. Our experimental results reveal that within the temperature range of 25 °C to 40 °C, viscoelasticity exhibits an upward trend. This observed enhancement with temperature can be partially attributed to the modifications in the degree of collagen alignment, which are dependent on the polymerization temperature [40,41]. The temperature range of 30–40 °C marks the initial thermal destabilization of the collagen structure in the cornea. This transition is characterized by a decrease in stiffness and an increase in viscosity, as indicated by the Brillouin spectral parameters. These changes reflect the beginning of structural modifications within the collagen network, leading to observable differences in the viscoelastic properties of the corneal tissue. Specifically, collagen matrices that were polymerized at different temperatures of 25 °C and 37 °C exhibited variations in alignment. This finding aligns with the work of Taufalele et al. [42], who demonstrated that polymerization temperature can modify the degree of collagen alignment. Such alterations in the matrix structure could potentially enhance the efficiency of acoustic wave transmission through the tissue matrix. Viscoelastic relaxation phenomena, inherent to the cornea’s biomechanical behavior, are also temperature-dependent [31]. Elevated temperatures may reduce the viscous component of the tissue, diminishing the energy dissipation and allowing sound waves to propagate more rapidly. Changes in the cornea’s hydration level with temperature can significantly influence hypersound velocity. As the cornea is heated, alterations in water content and distribution could account for the velocity increase, considering water’s high sound speed [43]. However, since we used silicone oil, there is little effect of hydration. Further studies are warranted to elucidate the precise microstructural alterations that contribute to the thermal behavior observed in our BLS measurements.

Throughout the temperature range of 40 °C to 60 °C, the corneal elasticity exhibits overall stability, suggesting resilience to temperature-induced changes. This consistency aligns with the cornea’s role in maintaining ocular integrity under various environmental conditions [44]. The minor changes can provide insights into the conformational changes that collagen fibrils experience. These changes are due to the disruption of various cross-links at the molecular level. This includes the nonenzymatic glycosylation of lysine and hydroxylysine residues at the intermolecular level and the breaking of disulfide bridges at the intramolecular level [45].

There are several limitations in this study. First, the choice of a 3-min acquisition time per spectrum was strategically made to balance signal integrity with practical constraints. This duration was primarily selected to minimize thermal drift and potential photodamage to the sensitive corneal tissues, which are common issues in prolonged Brillouin light scattering measurements. While shorter acquisition times can potentially reduce the signal-to-noise ratio, they significantly mitigate the risk of sample degradation due to extended exposure to laser illumination. Our trials indicated that extending the acquisition beyond three minutes offered minimal improvements in data quality but increased the risk of altering the sample properties, thus influencing the experimental outcomes. Future studies may explore alternative strategies to optimize acquisition time against signal quality, particularly as advancements in spectroscopic technology may allow for enhanced data collection efficiencies without compromising sample integrity.

Second, this study employs a simplified constant intensity background model in Brillouin scattering analysis, which, while effective for emphasizing the viscoelastic properties of corneal tissues at varying temperatures, may not fully capture the nuanced effects of Rayleigh scattering differences between silicone oil and corneal tissue. Our experimental design and data acquisition parameters, optimized for assessing biomechanical changes rather than dynamic scattering properties, limit the precision of detecting subtle tissue behaviors under thermal stress. Future research should consider more complex background models that account for these scattering differences to enhance measurement accuracy and provide a deeper understanding of tissue properties under varied conditions.

Third, in this study, isotropy was assumed in the corneal tissues based on their apparent homogeneity. However, corneal tissues are inherently anisotropic [46] due to variations such as collagen fiber orientation, a factor that can significantly influence the index of refraction and, subsequently, the Brillouin shift. Recognizing this, future studies will employ polarization-resolved Brillouin measurements to investigate these anisotropic properties more thoroughly. This limitation underscores the need for more detailed structural analysis to assess the biomechanical properties of the cornea accurately.

## 4. Conclusions

In this study, we applied Brillouin light scattering (BLS) spectroscopic sensing to assess the GHz-frequency viscoelastic properties across phase transitions of the ovine cornea, submerged in silicone oil to ensure consistent hydration over temperatures ranging from 25 to 70 °C. Through our analysis, we delineated heat-driven phase transitions within the physiological temperature range of 30–40 °C, attributed to alterations in molecular dynamics and intermolecular bonding within the collagen network. This transition precedes a more distinct phase shift observed at 60–63 °C, marked by significant decreases in Brillouin shift (stiffness), line width (viscosity), and scattered light intensity (elasto-optic interaction), indicative of collagen fibril denaturation and the consequential breakdown of the corneal tissue’s structured integrity. The use of silicone oil for hydration maintenance of the cornea was crucial in isolating the heating effect on the viscoelasticity of the cornea from that of the silicon oil detected by BLS sensing.

Our findings reveal the viscoelastic properties and phase behavior of corneal stroma and thereby enhance the biomedical understanding of the corneal response to thermal stress. Importantly, this study underscores the utility of BLS spectroscopy as a powerful bio-mechanical sensing technique for noninvasive, localized measurements of biopolymers such as corneal collagen. The ability to monitor optically and characterize these phase transitions in the cornea with sustained hydration within immiscible surrounding medium at both physiological and elevated temperatures can be used to implement in situ real-time noninvasive optical bio-sensing [35] of mechanical properties of the cornea during its refractive surgery.

## Data Availability

The data are available by requesting the authors.

## Appendix B

**Table A1:** Temperature Dependence of Standard Fitting Error and Standard Measurement Error.

Temperature (Celsius)	Standard Fitting Error	Standard Measurement Error
25	0.06	0.59324
28	0.07	0.56231
31	0.14	0.38672
34	0.09	0.42943
37	0.09	0.34161
40	0.15	0.32695
43	0.1	0.41276
46	0.14	0.53032
49	0.12	0.4497
52	0.17	0.4606
55	0.08	0.64402
58	0.2	0.84993
61	0.11	0.71722
64	0.14	0.75961
